# Favorable resuscitation characteristics in patients undergoing extracorporeal cardiopulmonary resuscitation: A secondary analysis of the INCEPTION-trial

**DOI:** 10.1016/j.resplu.2024.100657

**Published:** 2024-05-13

**Authors:** Johannes F.H. Ubben, Samuel Heuts, Thijs S.R. Delnoij, Martje M. Suverein, Renicus C. Hermanides, Luuk C. Otterspoor, Carlos V. Elzo Kraemer, Alexander P.J. Vlaar, Joris J. van der Heijden, Erik Scholten, Corstiaan den Uil, Dinis Dos Reis Miranda, Sakir Akin, Jesse de Metz, Iwan C.C. van der Horst, Bjorn Winkens, Jos G. Maessen, Roberto Lorusso, Marcel C.G. van de Poll

**Affiliations:** a Department of Anesthesiology and Pain Medicine, Maastricht University Medical Center (MUMC+), Maastricht, the Netherlands; b Cardiovascular Research Institute Maastricht (CARIM), Maastricht University, Maastricht, the Netherlands; c Department of Cardiothoracic Surgery, Maastricht University Medical Center (MUMC+), Maastricht, the Netherlands; d Department of Intensive Care Medicine, Maastricht University Medical Center (MUMC+), Maastricht, the Netherlands; e Department of Cardiology, Maastricht University Medical Center (MUMC+), Maastricht, the Netherlands; f Department of Cardiology, Isala Clinics, Zwolle, the Netherlands; g Department of Intensive Care Medicine, Catharina Hospital, Eindhoven, the Netherlands; h Department of Intensive Care Medicine, Leiden University Medical Center, Leiden, the Netherlands; i Department of Intensive Care Medicine, Amsterdam University Medical Center, Amsterdam, the Netherlands; j Department of Intensive Care Medicine, University Medical Center Utrecht, Utrecht, the Netherlands; k Department of Intensive Care Medicine, Sint Antonius Hospital, Nieuwegein, the Netherlands; l Department of Intensive Care Medicine, Erasmus Medical Center, Rotterdam, the Netherlands; m Department of Intensive Care Medicine, Maasstad Ziekenhuis, Rotterdam, the Netherlands; n Department of Cardiology, Erasmus Medical Center, Rotterdam, the Netherlands; o Department of Intensive Care Medicine, Haga Ziekehuis, The Hague, the Netherlands; p Department of Intensive Care Medicine, Onze Lieve Vrouwe Gasthuis, Amsterdam, the Netherlands; q Department of Methodology and Statistics, Maastricht University, Maastricht, the Netherlands; r Care and Public Health Research Institute (CAPHRI), Maastricht University, Maastricht, the Netherlands; s School for Nutrition and Translational Research in Metabolism (NUTRIM), Maastricht University, Maastricht, the Netherlands; t Department of Surgery, Maastricht University Medical Center (MUMC+), Maastricht, the Netherlands

**Keywords:** ECPR, Refractory Arrest, Ventricular Arrhytmias, OHCA, Resuscitation, Prognostic factors

## Abstract

**Introduction:**

Extracorporeal cardiopulmonary resuscitation (ECPR) is increasingly used as a supportive treatment for refractory out-of-hospital cardiac arrest (OHCA). Still, there is a paucity of data evaluating favorable and unfavorable prognostic characteristics in patients considered for ECPR.

**Methods:**

We performed a previously unplanned post-hoc analysis of the multicenter randomized controlled INCEPTION-trial. The study group consisted of patients receiving ECPR, irrespective of initial group randomization. The patients were divided into favorable survivors (cerebral performance category [CPC] 1–2) and unfavorable or non-survivors (CPC 3–5).

**Results:**

In the initial INCEPTION-trial, 134 patients were randomized. ECPR treatment was started in 46 (66%) of 70 patients in the ECPR treatment arm and 3 (4%) of 74 patients in the conventional treatment arm. No statistically significant differences in baseline characteristics, medical history, or causes of arrest were observed between survivors (*n* = 5) and non-survivors (*n* = 44). More patients in the surviving group had a shockable rhythm at the time of cannulation (60% vs. 14%, *p* = 0.037), underwent more defibrillation attempts (13 vs. 6, *p* = 0.002), and received higher dosages of amiodarone (450 mg vs 375 mg, *p* = 0.047) despite similar durations of resuscitation maneuvers. Furthermore, non-survivors more frequently had post-ECPR implantation adverse events.

**Conclusion:**

The persistence of ventricular arrhythmia is a favorable prognostic factor in patients with refractory OHCA undergoing an ECPR-based treatment. Future studies are warranted to confirm this finding and to establish additional prognostic factors.

**Clinical trial Registration:**clinicaltrials.gov registration number NCT03101787

## Introduction

Extracorporeal cardiopulmonary resuscitation (ECPR) is a therapeutic intervention that may prove helpful in augmenting conventional cardiopulmonary resuscitation (CCPR) for patients experiencing refractory out-of-hospital cardiac arrest (OHCA). Several randomized trials have been conducted during the past years, with differing results.^1^, ^2^, ^3^ These trials had different designs,^4^ varying from single-1, 2 to multicenter trials,^3^ and differed in their randomization strategy, which could be performed during the in-^1^ or pre-hospital phase.^2^, ^3^ Particularly, the latter feature may lead to the initial inclusion of patients that will still achieve return of spontaneous circulation (ROSC) in the pre-hospital phase, which might obscure the observed ECPR treatment effect given its favorable outcome.^5^, ^6^

Additionally, the Prague OHCA trial included patients with both shockable and non-shockable rhythms, while the ARREST and INCEPTION-trials only included patients with shockable rhythms. Based on prior registry data,^7^, ^8^ and a secondary post-hoc analysis of the Prague OHCA trial,^9^ particularly patients with shockable rhythms seem to benefit from ECPR, given the dismal prognosis of patients with non-shockable rhythms. Still, further investigations into favorable and unfavorable resuscitation characteristics are yet to be conducted. The potential recognition of such features may aid in the risk assessment or even in the decision-making process of patients with refractory OHCA who are being considered for this highly invasive treatment.

Therefore, the current secondary analysis of the INCEPTION-trial aims to identify potentially important prognostic resuscitation characteristics in patients receiving ECPR.

## Methods

### Trial design and ethical approval

The INCEPTION-trial was a Dutch multicenter randomized controlled trial (clinicaltrials.gov registration number NCT03101787). The research protocol was approved by the leading centers’ institutional review board (Maastricht University Medical Centre+ [NL58067.068.16/METC162039]) and adhered to the 2010 CONSORT statement.^10^ The concept of deferred consent was applied.^11^ A more comprehensive overview of the protocol, design, and informed consent procedure can be found elsewhere.^12^, ^13^

### Study setting

The INCEPTION-trial was performed in 10 Dutch centers that regularly provide extracorporeal membrane oxygenation (ECMO) therapy. These 10 centers cover a population of approximately 8 million inhabitants and are served by 12 emergency medical services (EMS). The study inclusion was performed between May 2017 and February 2021, with a temporary suspension of inclusion during the first SARS-CoV-2 wave.^13^

### Patients

For the study’s primary analysis (i.e., the intention-to-treat population), patients aged between 18 and 70 years suffering a witnessed OHCA with refractory ventricular arrhythmia (ventricular fibrillation, pulseless ventricular tachycardia, or a “shockable” rhythm detected by an AED) were eligible for inclusion. Refractory OHCA was defined as 15 min without return of spontaneous circulation (ROSC) despite advanced life support (ALS). Exclusion criteria were: stable ROSC within 15 min of the arrest; terminal heart failure (NYHA III or IV); chronic obstructive pulmonary disease (COPD) GOLD III or IV; oncological disease; pregnancy; bilateral femoral bypass surgery; pre-arrest Cerebral Performance Category (CPC) score of >2; multiple trauma (Injury Severity Score >15); an advance health care directive prohibiting resuscitation or invasive ventilation; an estimated time to start of cannulation longer than 60 min after the initial arrest.^12^ A refractory arrest was defined as 15 min of ALS without ROSC.

The current post-hoc analysis, which focuses on ECPR patients only, included all patients successfully treated with ECPR, irrespective of initial group randomization (cross-overs from CCPR to ECPR are also included in this analysis).

### ECPR implantation

Implantation of ECLS was performed in the emergency department (ED) or cardiac catheterization laboratory according to the specific centers’ protocol, either by the intensivist, interventional cardiologist, or cardiothoracic surgeon. The ECLS circuit [Cardiohelp System and HLS Sets Advanced 7.0 (Getinge)] was implanted in the femoral vessels percutaneously or by surgical cut-down.

### Outcomes

The current study focuses on identifying favorable pre-hospital resuscitation characteristics in patients considered for ECPR. A favorable outcome was defined as 30-day neurologically favorable survival. Neurologically favorable survival was defined as a cerebral performance category (CPC) of 1 or 2.^3^, ^12^, ^14^ Neurologically unfavorable survival was defined as CPC 3–5. Consequently, patients were divided into survivors (CPC1-2) and non-survivors (CPC 3–5), irrespective of vital status.

### Statistical analysis

The original trial was powered for the intention-to-treat population, and no specific sample size calculation was performed for the current secondary analysis.

Categorical data are summarized as numbers and percentages. Numerical variables were summarized by the median and interquartile range (IQR) or mean and standard deviation (SD). Between-group comparisons of numerical variables were performed using the Mann-Whitney *U* test if data did not follow a normal distribution or an unpaired *T*-test when data was normally distributed. The data was tested for normality using Shapiro-Wilk’s test, and P-P plots were visually assessed to evaluate the distribution. Categorical data were compared using the Chi-square test with Yates’ continuity correction and with Fisher’s exact test in case of an expected cell count < 5 (one-sided *p*-values were doubled to obtain a two-sided *p*-value when using Fisher’s exact test, as proposed previously^15^). A *p*-value below 0.05 was considered statistically significant for all analyses. Given the relatively small sample size and subsequent events, we did not construct an adjusted multivariable regression model.

Analyses were performed using SPSS (IBM Corp., version 27, Armonk, NY, USA).

### Role of the funding source

Getinge (commercial) and ZonMw (governmental) provided financial support to conduct the study. These providers did not have any influence on the study design, data collection, data analysis, interpretation of the data, drafting of the manuscript, or decision to submit.

## Results

### Patient inclusion

**Fig. 1:**
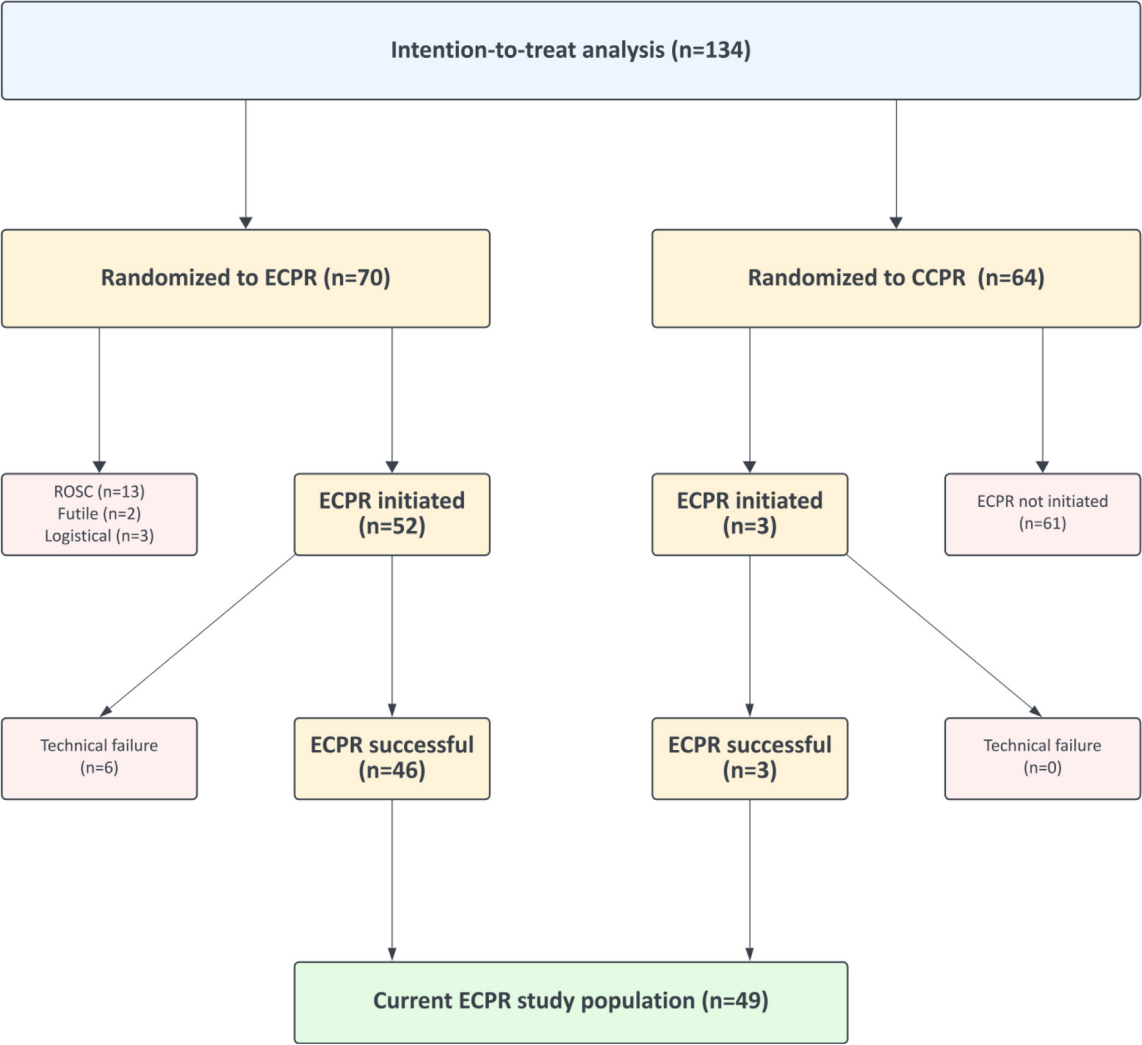
Flowchart for patient inclusion in this secondary analysis of the INCEPTION-trial.

Fig. 1 presents the flow of inclusion of patients for the current analysis. Of the 52 patients in which ECPR was initiated, six technical failures occurred (inability to advance arterial cannula [*n* = 1], inability to advance guidewire [*n* = 1], both cannulas in venous vessels [*n* = 1], inability to reach a flow > 0.5 L/min [*n* = 1], reason not registered [*n* = 2]). Notably, ECPR patients were not only derived from the initially randomized ECPR group with successful ECPR initiation (*n* = 46), but could also be cross-overs from patients randomized to conventional CPR (*n* = 3). Consequently, 49 patients were included. These patients were divided into 2 categories: survivors (5 patients) and non-survivors (44 patients).

### Patient characteristics

The survivors had a median age of 52 years [IQR 43–60 years] and non-survivors of 56 [47–65 years] (*p* = 0.449). All survivors were male (*n* = 5), while 91% of non-survivors were male (*n* = 40, *p* = 1.000). There were no significant differences in other baseline characteristics or medical history between survivors and non-survivors.

### Resuscitation characteristics

Survivors of ECPR received statistically significantly more defibrillation attempts than the non-survivors (13 [3,11–14] vs 6 [2–10], *p* = 0.002). A shockable rhythm was more frequently recorded as the last reported rhythm in the survivors (60% vs 14%, *p* = 0.037). The causes of arrest are reported in Table 1 and did not differ significantly between survivors and non-survivors (predominantly acute myocardial infarction). The dosages of medication given during the resuscitation attempts are described in Table 1 as well. The amiodarone dosage was significantly higher in survivors compared to non-survivors (450 mg [450–450 mg] vs 375 mg [0–450 mg], *p* = 0.047), while no other differences in dosages or laboratory values were observed.

**Table 1:** Baseline characteristics of survivors (CPC1-2) and non-survivors (CPC3-5).

	Survivors (*n* = 5)	Non-survivors (*n* = 44)	*p*-value
Demographics			
Age – yr.	52 [43–60]	56 [47–65]	0.449
Male sex – no. (%)	5 (100%)	40 (91%)	1.000
Primary shockable rhythm – no. (%)	5 (100%)	44 (100%)	1.000
Location of arrest: at home – no. (%)	2 (40%)	19 (43%)	1.000
Witnessed arrest – no. (%)	5 (100%)	44 (100%)	1.000
Total number of defibrillations	13 [3,11–14]	6 [2–10]	0.002
Transport distance − km	9 [3–21]	19 [3,7–23]	0.324
Last recorded rhythm			0.037
Shockable	3 (60%)	6 (14%)	
Non-shockable	2 (40%)	38 (86%)	
Cause of arrest			0.454
Acute myocardial infarction – no. (%)	4 (80%)	34 (77%)	
Secondary arrhythmia – no. (%)	1 (20%)	5 (11%)	
Pulmonary embolus – no. (%)	0	1 (2%)	
Metabolic/electrolyte – no. (%)	0	0	
Neurologic – no. (%)	0	1 (2%)	
Intoxication – no. (%)	0	1 (2%)	
Other – no. (%)	0	2 (5%)	
Medical history			
Acute coronary syndrome – no. (%)	0	7 (16%)	0.892
Coronary artery disease – no. (%)	1 (20%)	6 (14%)	1.000
PCI – no. (%)	1 (20%	2 (5%	0.562
CABG – no. (%)	0	2 (5%)	1.000
Chronic heart failure – no. (%)	0	4 (9%)	1.000
Cerebrovascular accident – no. (%)1	0	1 (2%)	1.000
Peripheral artery disease – no. (%)	0	1 (2%)	1.000
Diabetes Mellitus – no. (%)	0	8 (18%)	0.786
Hypertension – no. (%)	1 (20%)	15 (34%)	0.936
Hypercholesterolemia – no. (%)	0	7 (16%)	0.892
Current smoker – no. (%)	1 (20%)	14 (32%)	1.000
Pharmacological dosages and laboratory values			
Epinephrine dose – mg	9 [9,10]	10 [7–12]	0.283
Amiodarone dose – mg	450 [450–450]	375 [0–450]	0.047
pH – arterial	6.80 [6.78–6.82]	6.85 [6.80–7.01]	0.439
Lactic acid – mmol/L	17.4 [13.4–17.7]	13.7 [10.9–16.2]	0.534
Median partial pressure of carbon dioxide – kPa	11.8 [10.4–12.5]	10.1 [7.6–12.6]	0.778
Median partial pressure of oxygen – kPa	6.9 [4.2–7.7]	7.6 [2.4–11.8]	0.678

Values are medians with corresponding interquartile ranges. Percentages are rounded and may not add up to 100.

CABG: coronary artery bypass grafting. CPR: cardiopulmonary resuscitation, ED: emergency department, PCI: percutaneous coronary intervention.

Other resuscitation characteristics and ECPR are presented in Table 2, including differences in time intervals between survivors and non-survivors. There were no statistically significant differences regarding these durations. Of note, none of the patients randomized to CCPR who crossed over to ECPR ultimately survived.

**Table 2:** Resuscitation characteristics and ECPR intervals between survivors (CPC1-2) and non-survivors (CPC3-5).

Intervals	Survivors (*n* = 5)	Non-survivors (*n* = 44)	*p*-value
ROSC at ED arrival – no. (%)	0	0	NA
Intermittent ROSC – no. (%)	3 (60%)	19 (43%)	0.800
Start of arrest to EMT arrival – minutes	8 [7–12]	9 [6–10]	0.761
Start of arrest to EMT departure – minutes	35 [26–35]	28 [24–32]	0.308
Start of arrest to randomization – minutes	44 [37–45]	41 [32–48]	0.885
Start of arrest to ED arrival – minutes	43 [35–51]	46 [36–51]	1.000
Start of arrest to start cannulation – minutes	55 [49–60]	58 [52–66]	0.619
Hospital arrival to start cannulation – minutes	18 [14–17]	16 [3,12–22]	1.000
Start of arrest to start ECLS flow – minutes	70 [60–77]	74 [64–87]	0.712
Cannulation duration – minutes	11 [3,10–21]	19 [3,7–24]	0.761
Cannulation location ED – no. (%)	3 (60%)	32 (73%)	0.891
Cannulation location CCL – no. (%)	2 (40%)	12 (27%)	0.891

CCL: cardiac catheterization lab, ECLS: extracorporeal life support, ED: emergency department, EMT: emergency medical team.

### In-hospital outcomes and adverse events

In-hospital outcomes of survivors and non-survivors are described in Table 3. Percutaneous coronary interventions (PCIs) were performed in both survivors and non-survivors, and the frequency did not differ significantly between both groups. The duration of intensive care unit stay was significantly longer in survivors as compared to non-survivors (3 [2–5] days vs 1[0–4] days). The total duration of hospitalization was significantly longer in survivors of ECPR (18[3,9–27] days vs 2[0–8] days) as well. In-hospital serious adverse events were reported in Table 4.

**Table 3:** In-hospital outcomes of survivors (CPC1-2) and non-survivors (CPC3-5).

Outcomes	Survivors (*n* = 5)	Non-survivors (n = 44)	*p*-value
PCI performed – no. (%)	4 (80%)	25 (60%)	0.623
Admission to ICU – no. (%)	5 (100%)	40 (91%)	1.000
Duration of ICU stay (IQR) – days	3 [2–5]	1 [0–4]	0.004
Duration of hospitalization (IQR) – days	18 [3,9–27]	2 [0–8]	<0.001
Time from arrest to death after ICU admission – days	NA	1 [0–1]	NA

CCL: cardiac catheterization laboratory, ECLS: extracorporeal life support, ED: emergency department, ICU: intensive care unit, IQR: OR: odds ratio, interquartile range, PCI: percutaneous coronary intervention, ROSC: return of spontaneous resuscitation.

**Table 4:** In-hospital serious adverse events.

SAEs	Survivors (*n* = 5)	Non-survivors (*n* = 44)	*p*-value
Median number of SAEs per patient	0 [0–2.5]	1 [1,2]	0.249
Persisting cardiac failure – no. (%)	0	19 (43%)	0.149
Myocardial infarction – no. (%)	0	6 (14%)	1.000
Major bleeding – no. (%)	1 (20%)	10 (23%)	1.000
Infection – no. (%)	2 (40%)	2 (5%)	0.093
Post-anoxic encephalopathy – no. (%)	0	23 (52%)	0.069
Limb ischemia – no. (%)	1 (20%)	3 (7%)	0.719
Cannulation dislocation – no. (%)	0	4 (9%)	1.000
ELCS circulation failure – no. (%)	0	3 (7%)	1.000

ECLS: extracorporeal life support, SAE: serious adverse event.

## Discussion

The present study represents a secondary analysis of the INCEPTION-trial, which investigates the disparities in resuscitation characteristics among individuals who have experienced refractory OHCA and have been treated with ECPR. The analysis revealed that survivors were more likely to have persisting shockable rhythms and consequently received more defibrillation attempts with higher total amiodarone dosages compared to non-survivors. Therefore, the persistence of ventricular arrhythmia seems a favorable prognostic factor, in contrast to the conversion to a non-shockable rhythm.

Of note, survivors had a longer stay in the intensive care unit and hospital in the current study. This is the inherent consequence of a survivorship bias, which does not apply to non-survivors with a futile prognosis and short duration of ECPR.

### Shockable versus non-shockable rhythms

Survivors treated with ECPR had more frequent ventricular arrhythmia as the last reported rhythm. The total defibrillation attempts were more frequent in survivors than in non-survivors, and as a consequence of persistent ventricular arrhythmias, the total dosage of amiodarone was significantly higher. Although one may argue that these findings could be the consequence of the duration of CPR and subsequent ECPR (i.e., shorter duration to [E]CPR in the survivor-group), there were no significant differences in time spans between survivors and non-survivors. Our observations are in line with findings from the Minnesota ECPR program, which found a shockable presenting rhythm to be an important prognostic factor using a machine learning algorithm, in addition to intermittent ROSC, baseline lactate concentrations and arrest-to-perfusion time.^16^ In addition, Pozzi et al. even found that the persistence of shockable rhythms was the only independent prognostic predictor for neurologically favorable survival.^17^ A recent retrospective single-center analysis described cardiac rhythm changes during transport to the emergency department in OHCA patients.^18^ A significantly lower emergency department survival was observed in patients with cardiac rhythm change versus patients without cardiac rhythm change (26.5% vs 78.5%, *p* < 0.01).^16^ Three different categories of cardiac rhythm change appeared most frequently in the rhythm change group: ROSC to non-shockable (57.1%), shockable to non-shockable (26.5%), and non-shockable to ROSC (8.2%).^16^ Although this patient population differed from our study (as this study included all OHCA patients regardless of duration of arrest and rhythm), a significant number of patients in the rhythm conversion group had a conversion of a shockable to a non-shockable rhythm and a subsequent decline in their survival rate. Furthermore, another recent study demonstrated that a rhythm conversion from a non-shockable to a shockable rhythm resulted in improved survival, as compared to a persisting non-shockable rhythm.^19^ Indeed, primary shockable rhythms have been associated with improved survival as the underlying causes are more frequently reversible.^20^ The reason for the prognostic advantage of a persisting shockable rhythm cannot be drawn from our trial or the referenced studies. However, we can hypothesize that this may be the consequence of a more favorable CPR course and subsequently improved coronary perfusion during CPR, as depletion of myocardial adenosine triphosphate (ATP) reserve was associated with a decreased defibrillation success in animal models.^21^, ^22^

### Amiodarone dose and outcome

Administration of amiodarone in patients with cardiac arrest and a shockable rhythm is recommended in current guidelines.^23^ Despite its incorporation in international treatment guidelines, the evidence base for using amiodarone is not very strong. A recent double-blind randomized trial conducted in the US failed to show a beneficial effect on survival with favorable neurological outcome (modified Rankin scale > 3) after administration of amiodarone compared to lidocaine or placebo.^24^ However, the combined active drug arm (lidocaine or amiodarone as compared to placebo) was associated with a higher rate of survival to hospital discharge in patients with bystander arrest. Therefore, a secondary analysis of this study population was conducted, which revealed that early administration of amiodarone (within 8 min of arrival of emergency medical services) is associated with superior survival compared to placebo.^25^ This effect was not observed in patients receiving lidocaine administration, as compared to placebo.^25^ Another secondary analysis of this trial revealed a time-dependent decrease in survival with favorable neurological outcome when the time to active drug administration was extended.^26^ Although both ECPR and CCPR patients received amiodarone in our study, which also extends to ECPR survivors and non-survivors, the amiodarone dosage was higher in the survivor group. Whether this is the consequence of a persisting shockable rhythm or an actual amiodarone effect remains to be confirmed.

### Low-flow time

In the current study, the time from the start of arrest until ECMO flow initiation, the so-called low-flow time, did not differ significantly between survivors and non-survivors. This finding contrasts with other reports. A recently published systematic review and meta-analysis performed by Mandigers et al. showed a rapid decline in short-term survival of ECPR and CCPR-shockable patients when low-flow time increased.^27^ The survival after 15 min of low-flow duration was 37.2% in the ECPR- and 36.8% in the CCPR-shockable group. A 5% absolute risk difference in survival outcome was observed, starting from 16.5 min between ECPR and CCPR-shockable, in favor of ECPR. This difference increased to 22.6% at 30 min and 18.8% at 60 min of low-flow duration. The decline in short-term survival in relation to low-flow duration in ECPR was slower than in CCPR. The reason for the difference in decline between ECPR and CCPR remains to be elucidated. One potential reason could be a more stable organ perfusion with ECLS flow compared to less favorable organ perfusion without extracorporeal support in patients achieving ROSC.

Several authors have reported an important time-effect, sometimes considered the ‘golden hour’ of ECPR.^28^ Indeed, Bartos and colleagues described a time-dependent survival benefit between 20–60 min of low-flow time, which disappeared beyond an hour.^29^ After this timespan, there was no further correlation between time and outcome. Therefore, one may assume that ECPR should be initiated as soon as possible within this ‘golden hour’. However, past this point, other prognostic factors play a more important role, such as the persistence of a shockable rhythm.

Interestingly, we did not observe statistically significant differences in pH- and lactate-levels upon admission to the ED between ECPR survivors and non-survivors. Still, in a multicenter cohort study, pre-ECPR and 24-hour lactate levels were identified as a prognostic marker for 1-year survival.^30^ Furthermore, in a recent post-hoc analysis of the Prague OHCA trial,^31^ serial lactate levels measured during the first 24 h following arrest were significantly correlated with favorable neurological outcome in patients undergoing ECPR. Although our findings seem to contradict this analysis, the first measured lactate levels in the Prague OHCA cohort did not differ significantly either upon admission between survivors and non-survivors (7.8 vs 9.7 mmol/L). As such, the evolution of lactate levels seems to determine the outcome. However, lactate levels in INCEPTION’s ECPR population were markedly increased compared to the Prague OHCA cohort (survivors 17.4 versus 7.8 mmol/L, non-survivors 13.7 versus 9.7 mmol/L, respectively).

### Strengths and limitations

The data for the current study were derived from a prospective randomized controlled trial with a low selection bias. Following our study protocol, we could not exclude patients with unfavorable CPR characteristics such as prolonged CPR duration, changes in cardiac rhythms, or biochemical profiles after randomization to ECPR. In addition, there was an exceptionally high rate of complete follow-up. Furthermore, the INCEPTION-trial was a pragmatic multicenter trial, and its results seem, therefore, generalizable to many practices.

Still, several limitations should be mentioned. First, the research question addressed in this study was not predefined in the protocol.^12^ In addition, the post-hoc character introduces bias, as groups were not randomized. Furthermore, only a limited number of patients were included in this analysis (i.e., only patients receiving ECPR, even including patients crossed over from the CCPR group [*n* = 3]), and therefore, the sample size is markedly reduced compared to the primary analysis of the INCEPTION-trial.^3^ This makes our analyses susceptible to a type II error. In addition, six patients in the initial ECPR group were not studied because of technical cannulation failure. Finally, only five patients survived in all patients undergoing ECPR, which makes inferences complicated. Due to this limitation, we were unable to carry out a multivariable analysis. Nonetheless, the substantial effect of the examined risk factors indicates that the persistence of a shockable rhythm as an advantageous prognostic factor seems reliable and consistent with prior research.

## Conclusion

In this post-hoc analysis of the INCEPTION-trial, the persistence of ventricular arrhythmia is a favorable prognostic factor in patients with refractory OHCA undergoing ECPR. As our findings should be interpreted within the limitations of a small sample size, future studies are warranted to confirm this finding and to identify additional prognostic factors that can enhance the risk assessment and decision-making process of patients with refractory OHCA who are being considered for ECPR.

## Funding

Getinge (commercial) and ZonMw (governmental) provided financial support to conduct the study. These providers did not have any influence on the study design, data collection, data analysis, interpretation of the data, drafting of the manuscript, or decision to submit.

## CRediT authorship contribution statement

**Johannes F.H. Ubben:** Writing – original draft, Methodology, Investigation, Formal analysis. **Samuel Heuts:** Writing – original draft, Methodology, Investigation, Formal analysis. **Thijs S.R. Delnoij:** Writing – review & editing, Validation, Formal analysis, Data curation, Conceptualization. **Martje M. Suverein:** Writing – review & editing, Validation, Formal analysis, Data curation, Conceptualization. **Renicus C. Hermanides:** Writing – review & editing, Validation, Data curation. **Luuk C. Otterspoor:** Writing – review & editing, Validation, Data curation. **Carlos V. Elzo Kraemer:** Writing – review & editing, Validation, Data curation. **Alexander P.J. Vlaar:** Writing – review & editing, Validation, Data curation. **Joris J. van der Heijden:** Writing – review & editing, Validation, Data curation. **Erik Scholten:** Writing – review & editing, Validation, Data curation. **Corstiaan den Uil:** Writing – review & editing, Validation, Data curation. **Dinis Dos Reis Miranda:** Writing – review & editing, Validation, Data curation. **Sakir Akin:** Writing – review & editing, Validation, Data curation. **Jesse de Metz:** Writing – review & editing, Validation, Data curation. **Iwan C.C. van der Horst:** Writing – review & editing, Validation, Supervision, Methodology. **Bjorn Winkens:** Writing – review & editing, Validation, Methodology, Formal analysis. **Jos G. Maessen:** Writing – review & editing, Validation, Supervision, Resources, Methodology, Conceptualization. **Roberto Lorusso:** Writing – review & editing, Validation, Supervision, Resources, Methodology, Conceptualization. **Marcel C.G. van de Poll:** Writing – review & editing, Writing – original draft, Validation, Supervision, Resources, Methodology, Investigation, Funding acquisition, Formal analysis, Conceptualization.

## Declaration of competing interest

The authors declare the following financial interests/personal relationships which may be considered as potential competing interests: ‘Roberto Lorusso reports consulting fees from Medtronic, LivaNova, Getinge, and Abiomed and participates in an advisory board of Eurosets and Xenios, which are not related to this work. All other authors report no conflicts of interest.’.
